# Simultaneous optimization of parameters influencing organic mulch test using response surface methodology

**DOI:** 10.1038/s41598-020-63047-y

**Published:** 2020-04-21

**Authors:** Saeed Shojaei, Mohammad Ali Hakimzadeh Ardakani, Hamid Sodaiezadeh, Mohammad jafari, Seyed fakhreddin afzali

**Affiliations:** 1 Department of Management Arid and Desert Regions, College of Natural Resources and Desert, Yazd University Yazd, Iran; 2 Department of Arid and Mountainous Regions Reclamation, Faculty of Natural Resources, University of Tehran, Tehran, Iran; 3 Department of Natural Resources and Environmental Engineering, College of Agriculture, Shiraz University Shiraz, Iran

**Keywords:** Environmental monitoring, Environmental impact, Natural hazards, Risk factors

## Abstract

Wind erosion could be regarded as one of the most important phenomena especially in arid lands in the globe, which destroys vast areas with its rapid growth. Due to global droughts and climate change, vegetation is weakened and soil particles are more easily exposed to wind erosion. Today, various methods have been developed to control wind erosion. One of the most commonly used methods is the use of mulch. Mulch has several types (physical-chemical). The purpose of this research is to make organic mulch with inexpensive and available compounds to prevent environmental pollution as well as preventing wind erosion. For this purpose, Chaff, Manure, Biosolids and Black Strap mixture were used. The surface response method (RSM) was utilized to perform the relevant tests and create optimal situation. In this study, the central composite design (CCD) in RSM modeling was applied to make the optimal experimental conditions of erosion and penetration resistance. The effect of concentration of Chaff, Manure, Biosolids and Black Strap, was evaluated in erosion and penetration resistance and each variable was coded at five levels. The optimum values for penetration resistance of 1.8 (kg/cm^2^) for Chaff, Manure, Biosolids, and Black Strap, were 11.32, 15.72, 19.23 and 4.37 g, respectively. Also, the optimum values of mulch combination for wind erosion of 1.8 kg/cm^2^ for Chaff, Manure, Biosolids, and Black Strap, were 12.49, 19.95, 16.71 and 7.15 g, respectively. By increasing the use of these compounds, adhesion and erosion resistance increased and more consumption of Black Strap had a reverse effect. This was due to the loosing of soil particles and creating exponential mode in graphs. According to the results, no particular distinction was found between predicted data by Design-Expert software and experimental data and simulation results can be expressed reliably.

## Introduction

One of the environmental problems in the arid and desert areas of the world that operates externally is the wind erosion and its consequences, such as dust storms and the displacement of massive masses of sand^1–3^. Dust storms destroy lands, reduce visibility (dust concentrations in some cases reach 6 g/m^3^), and cause the spread of radioactive materialals^4^. The Greenhouse Effect^5^ leads to the transport of salt and chemical substances (herbicides) to the atmosphere, and increases respiratory problems^6,7^. Over the half past century, various materials have been evaluated to find suitable stabilizers for controlling wind erosion^8^. One common method for controlling the movement of sand is mulching over its surface^1^. The word “mulch” in English means “cover”. These materials cover the soil surface as a thin coating and protect soil, water, and plants from damage. This term is more often referred to as soil cover by organic materials such as straw, chaff, plant leaves, and sometimes animal Manures and similar substances. Mulch refers to natural or artificial materials that protect the soil from the damage of various factors such as wind and rain^9^. Mulch reduces water evaporation from the soil surface by creating shadows and also preventing the movement of air on the ground. Therefore, it maintains soil moisture, prevents sudden changes in temperature and increases the fertilization and soil productivity. In general, the purpose of mulching in fixing sandy soils is to increase soil stability in order to provide an opportunity for the establishment of other biological activities such as planting and seeding, so that plant species can be located in the field of planting^9^. In different sources, different categorizations are presented for mulches In general, mulches can be divided into two physical and chemical groups^10^:

1. Physical mulches: These mulches include: (a) biological mulches: Straw and chaff and Manure (b) non-biological mulches: pebbles, solid wastes, clay and cement types.
2. Chemical mulches: These mulches include: (a) oil mulches.; (b) Non-oil mulches

Oil mulches: It is referred to oil products made of hydrocarbon mixtures and used to cement sand particles. However, due to high costs of oil materials and environmental issues, they are not used any more.

Non-oil mulches: include all materials or coatings used to prevent water evaporation, weed growth, and generally to increase soil yields. Non-oil mulches include various synthetic chemical polymers, lime, gypsum, and herbal gums.

Vaezi^11^ in an examination of the use of oil mulch in controlling wind erosion and stabilizing the sand, states that oil mulch is bitumen-like. Therefore, their dark color causes the area to be warmed up more than the surrounding environment, which eliminates the energy balance of the environment and causes the wind in the region. The use of biologic mulch increases the resistance to dust harvesting and surface resistance (penetration resistance). The penetrometer method is an appropriate method for testing mulch^12^. Soil biologic stabilizers have been considered as new stabilizers and have become increasingly important in recent decades. These stabilizers will allow the soil particles to stick together and maintain moisture. These materials are eco-friendly and have no impact on environment and are part of eco-friendly stabilizers^13^. Examples of stabilizers used to date include: cement mulch mixed with soil^14^, calcareous stabilizers and organic stabilizers^15^. These stabilizers, when mixed with soil, cause adhesion and cation exchange^16^. In researches conducted around the world, mulch tests have been performed as a randomized block and mulch application proposed by the expert without testing all of the interval to determine the amount of mulch use. For example, some researchers have proposed the use of 400 g Cement + 10 g Lime^14^, 250 gr Clay + 25 gr Straw^17^ (Majdi *et al*. (2005), 250 g Dunder^18^, which are examples of different researches in this field. There has not been any specific method to produce mulch, and all provided mulch types are mono structural and not optimized. Therefore, in this research, the purpose is to create mulch using new mixture and formula in order to manage the haze and dust pollution. The selected organic materials are mixed together by the software. Then, based on those results, the best formulation is determined for resistance to erosion and penetration. Considering the application of this new method, the results of this study will greatly help to reduce the damage caused by dust and haze pollution around the world. Is used as a new method.

## Materials and methods

**Figure 1:**
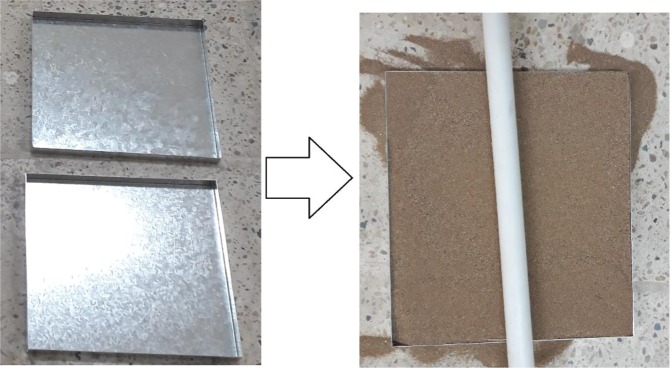
A sample of the trays used in this experiment.

This study was conducted at the research laboratory of Yazd University. In order to carry out this research, dune sand samples were taken from sandy hills (Yazd area) and transferred to the wind erosion laboratory and were used as a test bed and also were mixed with mulch. Then were spread onto the special trays^19^. These trays with dimensions of 25×25×2 cm were used in the wind tunnel. Then, we filled the trays with filtered sand using 2 mm sieve and also leveled out the tray surfaces through leveling rods and in the next step, we spread the blended mulch all over over the trays. The trays were made of metal (Fig. 1).

After determining the desired mulch composition, adding 500 mm of water to these compounds and mixing it completely, the mulch was prepared for spraying onto the trays. The mulch was sprayed on the tray with the aid of a sprinkler. The prepared trays were placed in free air for two days from the time of spraying, to dry completely. In this research, materials used to make mulch were Chaff, Manure, Biosolid and Black Strap.

### Tests

#### Wind tunnel Test

**Figure 2:**
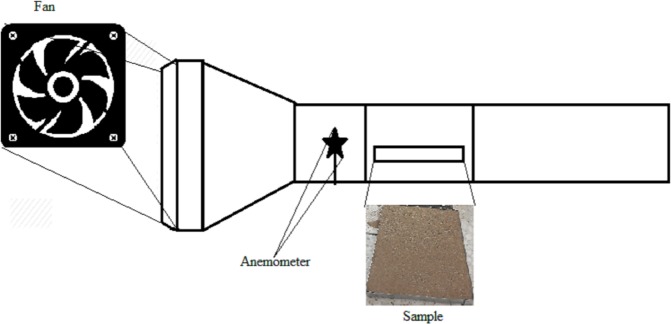
A wind tunnel with an open circuit system.

The wind tunnel has a blower with a capacity of 2030 m^3^/h, equivalent to 1,218 m^3^/min (CFM), 2300 RPM (Revolutions per minute), a current of 0.66 A, and a power of 145 W, which produces wind power with a maximum speed of 12.5 m.s^−1^. The motor is mounted on an MDF (Medium-Density Fibreboard) frame with a dimensions of 0.03 × 0.05 m which is attached to an MDF sheet with a dimension of 0.3 × 0.5 m. In order to prevent its possible slipping, two MDF sheets are attached from the top of the frame to the end of the sheet. The samples will be placed in the wind tunnel^20^ for 20 minutes at a speed of 12 m.s^−1^ at a height of 15 cm (Fig. 2).

#### Penetrometer test

The penetrometer is a device in which the pressure is applied on the surface of the soil with its 1 cm^2^ tip. When the tip is entered into the soil, the maximum pressure applied to the surface is displayed in kg/cm^2^. The penetrometer is used to test the compressive strength of the soil surface, the compressive strength of the crust formed on the soil surface, and the compressive strength of the mulch dispersed on the soil surface^21^.

### Response surface methodology (RSM

RSM is a way to study search optimum conditions for a multi-variable system which can optimize conditions. One of the most important advantages of RSM is to reduce the number of experiments and as a result, it could save costs and it would be less time-consuming. Also, there is the chance to analyze the effects of different factors over each other and changing trends. Since we need RSM to carry out the experiments, we have to know the design of experiment. The design of experiments involves a series of experiments that deliberately create changes in the process input variables, and in this way, the amount of resulting variation in the process output response is detected and identified^22^. The process can be thought of as a combination of devices, methods, and individuals that converts input materials into an output product^23^. This output product has one or more qualitative attributes or visible responses. Some process variables are controllable and others are uncontrollable (although they can be controlled in test conditions). In this method, the effect of all factors is considered. We used Design-Expert to design and analyze the experiment and the results are given to the software. It calculates one-way analysis of variance to check the preciseness of the experiment^24^. Then, the model and formulation are carried out. Finally, the software optimizes the factors in the experiment. All the results of penetrometer and wind tunnel tests were introduced into Design-Expert software. The design of experiments was done based on the number of treatment which are entered in the software and the data were given to the software. Then, based on the data in the experiment, the best mathematical model was chosen and then diagrams were illustrated, optimized and finally the formula was presented.

### Experimental design and data analysis

As the research tasks are developing, it is required to use some methods to reduce the number of experiments and reach the final results as quickly as possible. Therefore, a proper design for experiments can not only lower the costs, but also paves the way for more reliable results. An exact way to design such experiments is to use Design-Expert which has been applied in this research.

In this research, four independent variables including Chaff, Manure, Biosolide and Black Strap were examined and each variable was coded at five levels (Table 1). The test was designed by Design-Expert 10.0.830 software. Equation 1 was used to code variables:

1 $${{Z}}_{{i}}=\frac{{x}_{i}+{x}_{o}}{\Delta {{x}}_{{i}}}$$

where: In this relation, *Z*_*i*_ is the value of the encoded independent variable, *x*_*i*_ and *x*_0_ are the actual values of the independent variables, *x*_0_ is the central point, and *Δx*_*i*_ is amount of variation.

**Table 1:** Experimental range and levels of independent process variables.

Variables	Unit	Symbol coded	Levels				
			−2	−1	0	1	2
Chaff	g	*A*	4	5	6	7	8
Manure	g	*B*	12	14	16	18	20
Biosolids	g	*C*	12	14	16	18	20
Black Strap	g	*D*	7.5	8.5	9.5	10.5	12.5

The best method for assessing the fitness quality of the model is to use ANOVA analysis for test results^24^. After entering the results into Design-Expert software, the quadratic model was considered as the ideal model for resistance to penetration and wind erosion among different mathematical models. The quadratic equation is shown in Eq. (2):

2 $$Y={b}_{0}+\mathop{\sum }\limits_{i=1}^{4}{b}_{i}{x}_{i}+\mathop{\sum }\limits_{i=1}^{4}{b}_{ii}{{x}_{i}}^{2}+\mathop{\sum }\limits_{i=1}^{3}\mathop{\sum }\limits_{j=i+1}^{4}{b}_{ij}{x}_{i}{x}_{j}$$

where: *Y* is the predicted response, *b*_0_ is the coefficient of width from the origin, *bi* represents the linear coefficient of the parameters, *b*_*ii*_ is the second-order interaction coefficient, *b*_*ij*_ is the second-order coefficient, and *x*_*i*_ and *x*_*j*_ are independent encoded variables^25,26^. As shown in Table 2, the P-value is calculated for each model and the best model is also chosen based on the conditions.

**Table 2:** Statistical parameters for sequential models.

Source	Penetrometer					Wind Erosion					
	Sum of squares	df	Mean square	F-value	P-value	Sum of squares	df	Mean square	F-value	P-value	Remarks
Linear	0.22	20	0.019	40.05	<0.0001	160.600	4	30.170	17.555	<0.0001	—
2FI (two factor interactions)	0.20	14	0.016	1.10	0.04000	0.250	6	0.050	0.021	1.0000	—
Quadratic	0.9	10	0.07	5.00	0.0066	46.698	4	14.500	56.485	<0.0001	Suggested
Cubic	0.040	2	0.020	0.60	0.3547	2.368	8	0.590	4.585	0.0412	Aliased

## Results and discussion

### Statistical analysis

Experimental data are presented for the permeability and wind erosion of the produced mulch in Table 3. The final model for predicting the permeability and wind erosion is shown in Equations 3 and 4:

**Table:** 

Penetrometer=1.60 + 0.10 *A* + 0.1*B* + 0.10 *C* + 0.19*D* + 0.018*AB*−0.022*AC* + 0.022*AD*-0.0140*BC* + 0.0140*BD*-0.040*CD* + 3.10*A*^2^ + 3.10*B*^2^ + 0.030*C*^2^ + 0.075*D*^2^ (3)	*A*: Chaff *B*: Manure *C*: Biosolids *D*: Bla ck Strap
Wind tunnel=20-1.48*A*-0.2*B*-0.35*C*-2.55*D* + 0.14*AB* + 0.14*AC* + 0.63*AD* + 0.63*BC* + 0.07*BD* + 0.09*CD*-0.72*A*^2^ + 8.29*B*^2^-4.6*C*^2^-1.5*D*^2^ (4)

**Table 3:** Response surface central composite design and experimental and predicted responses.

Variables					Response			
					Penetrometer (kg/cm^2^)		Wind Erosion (g/m^2^ h)	
Run	*A*	*B*	*C*	*D*	Experimental	Predicted	Experimental	Predicted
1	5	14	14	8.5	1.1	1.23	24.60	24.66
2	7	14	14	8.5	1.6	1.60	21.99	22.20
3	5	18	14	8.5	1.5	1.49	24.20	26.80
4	7	18	14	8.5	1.7	1.60	21.00	20.70
5	5	14	18	8.5	1.7	1.63	23.10	22.00
6	7	14	18	8.5	1.6	1.71	20.97	20.88
7	5	18	18	8.5	1.9	1.79	22.40	22.00
8	7	18	18	8.5	1.8	1.87	18.90	19.15
9	5	14	14	10.5	1.6	1.61	18.50	18.60
10	7	14	14	10.5	2.1	2.00	15.60	15.70
11	5	18	14	10.5	2	1.85	18.15	18.30
12	7	18	14	10.5	2.3	2.27	15.12	15.44
13	5	14	18	10.5	2	1.97	17.42	17.90
14	7	14	18	10.5	2.1	2.15	15.80	15.09
15	5	18	18	10.5	2.1	2.11	16.30	16.63
16	7	18	18	10.5	2.5	2.45	15.00	14.60
17	4	16	16	9.5	1.5	1.50	22.00	23.22
18	8	16	16	9.5	2	1.97	15.10	15.80
19	6	12	16	9.5	1.5	1.50	22.20	22.05
20	6	20	16	9.5	2	1.97	20.31	20.70
21	6	16	12	9.5	1.7	1.59	21.50	21.55
22	6	16	20	9.5	2.2	2.18	20.15	20.45
23	6	16	16	7.5	1.5	1.42	18.30	19.67
24	6	16	16	12.5	2.3	2.22	11.42	10.76
25	6	16	16	9.5	1.6	1.60	20.00	21.00
26	6	16	16	9.5	1.5	1.60	20.00	21.00
27	6	16	16	9.5	1.6	1.60	20.00	21.00
28	6	16	16	9.5	1.6	1.60	20.00	21.00
29	6	16	16	9.5	1.6	1.60	20.00	21.00
30	6	16	16	9.5	1.6	1.60	20.00	21.00

The analysis of variance and factor values are displayed in Table 4. The calculated F-values for permeability and wind erosion (26.241 and 59.069 respectively) are more than the tabular values. The calculated R^2^ values for permeability and wind erosion are 0.9541 and 0.9710, respectively, which indicates the acceptable accuracy of the model in estimating the predicted data from the experimental data.

**Table 4:** Analysis of variance (ANOVA) results of quadratic model to Penetrometer and Wind Erosion.

Source	df	Penetrometer				Wind Erosion			
		Sum of squares	Mean square	F-value	P-value	Sum of squares	Mean square	F-value	P-value
Model	14	3.419	0.186	26.241	<0.0001	312.123	16.503	59.069	<0.0001
A	1	0.444	0.457	47.777	<0.0001	60.807	53.227	148.425	<0.0001
B	1	0.340	0.298	36.152	<0.0001	1.420	1.140	3.871	0.0601
C	1	0.530	0.381	63.469	<0.0001	2.697	2.819	9.206	0.0081
D	1	1.316	1.209	170.115	<0.0001	132.874	101.968	525.369	<0.0001
AB	1	0.003	0.003	0.342	0.5003	0.050	0.050	0.150	0.6521
AC	1	0.025	0.028	3.203	0.0885	0.050	0.050	0.150	0.6521
AD	1	0.025	0.028	3.113	0.0885	0.050	0.050	0.150	0.6521
BC	1	0.003	0.003	0.322	0.5963	0.050	0.050	0.150	0.6521
BD	1	0.025	0.027	0.300	0.5789	0.050	0.050	0.150	0.6521
CD	1	0.000	0.000	3.090	0.0888	0.050	0.050	0.150	0.6521
A^2^	1	0.026	0.023	0.060	0.7184	8.975	8.895	30.521	<0.0001
B^2^	1	0.140	0.162	0.060	0.7144	0.003	0.002	0.008	0.8882
C^2^	15	0.122	0.009	3.555	0.0854	0.000	0.000	0.001	0.8974
D^2^	10	0.113	0.012	16.650	0.0006	61.400	60.885	302.125	<0.0001
Residual	5	0.000	0.000			4.448	0.358		
Lack of Fit	29	2.733	0.143			4.258	0.436		
Pure Error	14	2.616	0.387			0.000	0.000		
Cor Total	1	0.344				250.700			
Model Summary Statistics									
Precision		Penetrometer					Wind Erosion		
		R^2^	Adj-R^2^	Adequate Precision			R^2^	Adj-R^2^	Adequate Precision
		0.9541	0.9200	21.521			0.9710	0.9600	30.514

The Adj-R^2^ values for permeability and wind erosion (0.9200 and 0.9600 respectively) confirms the proposed model. By measuring the signal-to-noise ratio, the accuracy of the model can be shown, which should be greater than 4. In this study, this ratio is 21.521 and 30.514 for the values of permeability and wind erosion, respectively which indicates a high degree of accuracy^24^.

**Figure 3:**
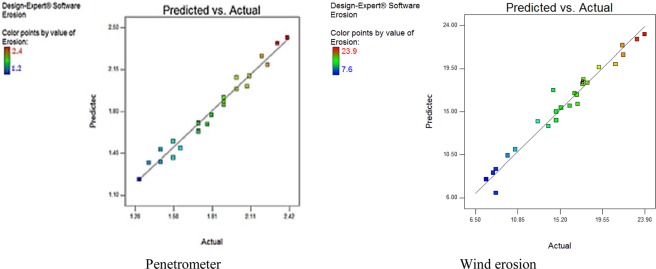
Scatter plot of predicted value vs actual value from RSM design.

Fig. 3 shows the results of the actual (experimental) values of penetrometer and wind tunnel against the predicted values of the response surface method. The values of the determination coefficient (R^2^) for pentameter and wind tunnel, are 0.9541 and 0.9710, respectively, which indicate the reasonable precision between the data and the model.

### Interaction between influencing factors

**Figure 4:**
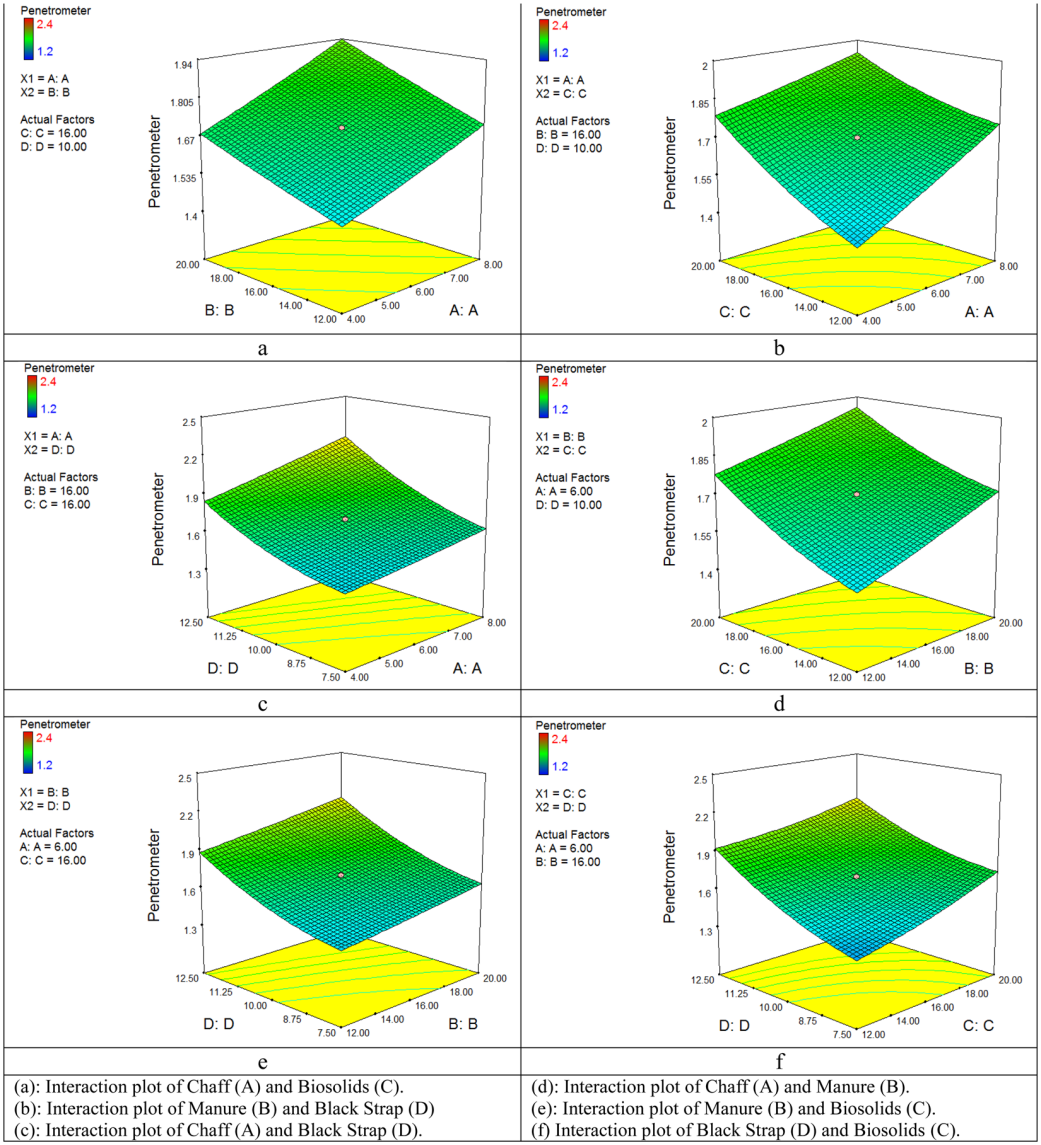
Response surfaces plot on Penetrometer.

The three-dimensional (3D) response surface plots were used to understand the interactions of the studied factors in mulch on the responses and relationships between them. Figure 4, the 3D response surface plots function was utilized to investigate the interaction between the materials used in mulch on the penetration resistance.

Soils that are stabilized with the combination of mineral mulches and organic matter (biopolymer) show higher resistance to storm and surface resistance (maximum penetration force) due to increased surface penetration (maximum penetration strength) and high adhesion. The penetrometer test is a suitable method for determining resistance to erosion^13^.

In Fig. 4(a–c), the interaction between Chaff with the Manure, Biosolids, and Black Strap content is investigated for resistance to permeability. As is shown, with increasing the amount of Chaff in the mulch, the resistance to penetration is also increased, and consequently, the direct effect is also observed. The effect of Blackstrap on the resistance to penetration in organic mulch was higher than Chaff, Manure, and Biosolids. If the amount of Chaff is considered constant, with an increase in the amount of Black Strap, the soil compaction was less. The reason for this is its fluidity property and the need for more time to dry.

Research has been conducted on the role of Chaff in permeability resistance. For example, researchers conducted experiments on the effect of Chaff mulch on the resistance to permeability in various soils of America. The amount of mulch used in different soils was two and a half tons of Chaff per acre. The results of the studies are shown that the difference between the resistance to permeability in soils with mulch and non-mulch is quite significant and comparable^13^.

In Fig. 4(a,d,e), the interaction of the Manure parameter with the Chaff, Biosolids, and Black Strap content is investigated for resistance to permeability. As Manure value is raised, the penetration resistance is increased, but this increase was low because, with increasing the amount of Manure in the mulch, the adhesion of soil particles is first increased and then decreased, and the resistance to penetration changed. The effect of Manure on the resistance to penetration in organic mulch is relatively similar to Chaff and have no significant effect on penetration resistance, but the effect of Manure on soil adhesion and penetration resistance were less than Biosolids and Black Strap. Black Strap leads to significant adhesion of soil particles.

In Fig. 4(b,d,f), the interaction of the Biosolids parameter with the Chaff, Manure, and Black Strap content are investigated for resistance to permeability. By increasing the Biosolids content, the resistance to penetration is increased. The effect of Biosolids on the resistance to penetration in the organic mulch is relatively similar to that of Chaff and Manure, but Black Strap leads to more adhesion of soil particles compared to Biosolids.

Arid soils often have the lowest amount of organic matter. Therefore, due to the limited resources of Manure, the use of other organic Manure sources, such as Biosolids, is desirable^21,23^. Sewage sludge is rich in plant required elements and is therefore considered to be a low-cost Manure by agricultural experts. Also, an increase of 5% Biosolids increased the hydraulic conductivity of soil in silt loam tissue^27^, as well as soil stability, improve specific mass and soil porosity is increased. By improving these factors, the strength of the soil surface layer is increased (penetration resistance) and the wind erosion is decreased^28^.

In Fig. 4(c,e,f), the interaction of the Black Strap parameter with Chaff, Manure, and Biosolids are investigated for resistance to permeability. By adding the value of Black Strap, the penetration resistance is increased due to the high adhesion properties of this material in compressing the soil. The effect of Black Strap on the resistance to penetration in organic mulch was higher than that of all used materials (Chaff, Manure, and Biosolids). Khalili Moghadam *et al*.^18^ conducted a study in Khuzestan with the aim of making mulch from sugar cane residues and comparing it with oil mulch. In this study, the composition of different percentages of sand and sugar cane residues was used. They selected seven compounds. This experiment was conducted as a factorial in a thoroughly randomized mode. In the laboratory, using the mulch spray, the shifting sands was sprayed on the bed. The shear strength and soil penetration resistance of each treatment were measured using a vane shear device. The results represented that by increasing the thickness of the mulch layer, the shear strength and penetration resistance is increased. The combination of 250 g of sugar cane residues has a better result.

**Figure 5:**
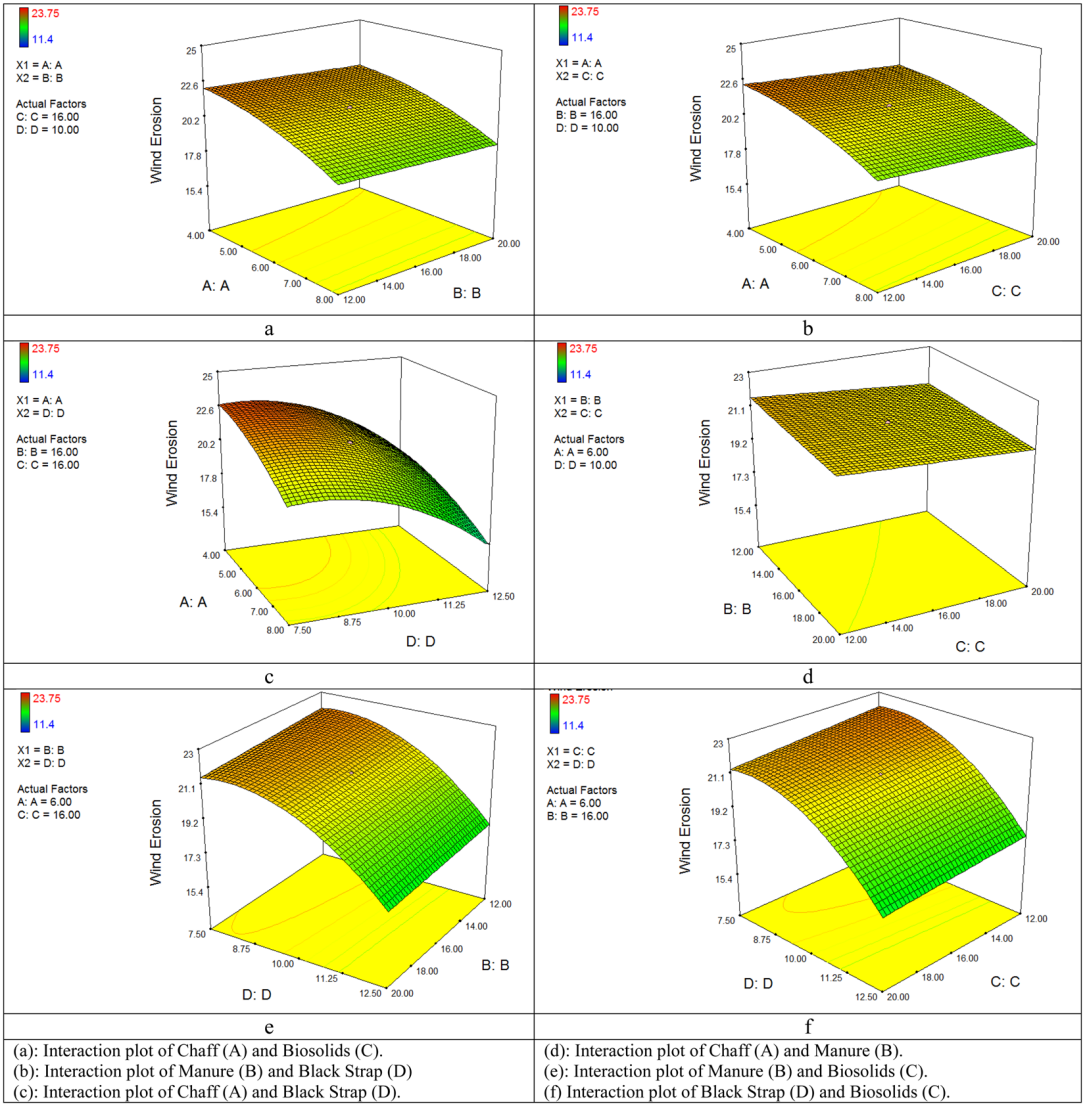
Response surfaces plot on Wind erosion.

In Fig. 5(a–c), the interaction of the Chaff parameters and Manure, Biosolids, and Black Strap content is investigated for wind erosion. As shown, with increasing the amount of Chaff in the mulch, the wind erosion is also decreased, and so direct effect is observed. The effect of Black Strap on wind erosion resistance in organic mulch was higher than that of Chaff, Manure, and Biosolids, and the rate of wind erosion is significantly reduced. If the amount of Chaff is considered constant, with an increase of the Black Strap, the wind erosion of the soil is decreased with a steep gradient.

Adelpor *et al*.,^29^ examined the effect of Straw application on erosion control in different regions. First, according to the amount of Straw, areas are divided into three regions: poor regions (less than 20% of Straw in the whole region), moderate (20-40% Straw in the whole region), and good areas (more than 40% of Straw in the whole region) in terms of plant residues. The results showed that lands with low Straw content have a significant soil resistance to erosion^28,30^.

In Fig. 5(a,d,e), the interaction of Manure parameters with Chaff, Biosolids, and Black Strap is investigated for the reduction of wind erosion. By increasing the amount of Manure, wind erosion is decreased, but this decrease was low because, by increasing the amount of Manure in the mulch, the adhesion of soil particles first is increased and then decreased and causes the change of wind erosion. The effect of Manure on the rate of wind erosion in organic mulch is relatively similar to that of Chaff and has no significant effect on reducing wind erosion, but the effect of Manure on reducing the wind erosion of soil particles compared to the Biosolids and the Black Strap was very low. Black Strap leads to significant adhesion of soil particles and reduced the wind erosion. The effect of Manure and Biosolids on the reduction of wind erosion was insignificant, which can be ignored (Fig. 5). In Fig. 5(b,d,f), the interaction of Biosolids with Chaff, Manure, and a Black Strap on the reduction of wind erosion are investigated. The results showed that by increasing the amount of Biosolids, the wind erosion did not change much. The effect of Biosolids on reducing wind erosion in an organic mulch is almost the same as that of Manure, but the changes in the reduction of wind erosion in the Chaff and the Black Strap were higher.

Hosseini *et al*.,^21^ investigated the capability and effectiveness of Manures as an anti-erosion agent. The results showed that soil treated with a mixture of water and Manure forms a shell on the soil surface. Two types of Manures (compost and Biosolids) were compared in two roughness of the soil surface (flat and rough). Wind erosion in non-treated soil at 6 m.s^−1^ wind speed began on both flat and rough soil surface, while after using the Manure, the wind erosion rate reached 14-12 m.s^−1^. There was no significant difference between the wind erosion of two Manures^21^.

In Fig. 5(c,e,f), the interaction of the Black Strap parameter with Chaff, Manure, and Biosolids on the reduction of wind erosion are investigated. By increasing the amount of Black Strap, the wind erosion is reduced. The reason for this is the high adhesion properties of this material, which creates a crust on the surface layer of the soil and reduced the wind erosion. The effect of the Black Strap on reducing the wind erosion in the organic mulch was higher than that of all used materials (Chaff, Manure, and Biosolids). Allahdady *et al*.,^31^ investigated the Black Strap resistance in particle adhesion and increased tensile strength and separation of particles from a number of natural substances (natural fibers, poly-latex, and sugar cane residues). They concluded that, given that the Black Strap has an adhesion property, it is shown a better result in terms of tensile strength, which is consistent with the findings of this study^31^.

Farahmehr *et al*.,^32^ investigated the clay-limestone mulch, sugar cane residues, gravels, steel slag, calcium and magnesium chloride, Poly-latise, and polyvinyl acetate for stabilizing shifting sands. These mulches were examined in laboratory and environment. Their results showed that chemical mulches, although have greater adhesion, resistance to penetration, and wind erosion, cause pollution of the environment. Organic mulches, however, can add nutrients to the soil, and they can be effective in soil stability against wind erosion^33^.

### Optimization of reaction

The main purpose of RSM is to choose factors on order to reach the maximum response. In numeral optimization, Design-Expert is used to analyze the factor and response. Each goal could be used by this software to maximize, minimize, and target in the studied span. In this study, in order to minimize the value of Chaff, Manure, Biosolids, and Black Strap and get the best results in terms of soil resistance and less wind erosion, we used optimization. It is possible to control the value of the materials through optimization which could be used in national and international projects. Therefore, after determining the effect of different factors on the penetration resistance of mulch and wind erosion, to determine the best efficiency for conditions (penetration resistance of mulch and wind erosion), certain levels of effective input parameters are selected. So, the ideal conditions for Chaff, Manure, Biosolids, and Black Strap are chosen without any restrictions on consumption. According to the results obtained by the software, three optimized combinations suggested by the software were chosen and then they were tested and evaluated again. The optimal values of the variables are presented in Tables 5 and 6. The maximum values for infiltration resistance for organic mulch for Chaff, Manure, Biosolids, Black Strap are 4.84 g, 18.33 g, 16.60 g, and 11.50 g respectively and the result was 90.80%. Moreover, the maximum values of erosion resistance in organic mulch for Chaff, Manure, Biosolids, and Black Strap are 8.18 g, 17.70 g, 18.90 g, and 13.90 g and the result was 91.40%.

**Table 5:** Optimum conditions derived by RSM for Resistance to Penetrometer.

Exp.	Optimal conditions				Penetrometer	
	*D*	*C*	*B*	*A*	Experimental (%)	Predicted (%)
1	11.50	16.60	18.33	4.89	90.80	92.50
2	9.56	20.11	16.54	8.20	90.40	91.30
3	12.20	20.00	15.70	6.69	90.20	90.10

**Table 6:** Optimum conditions derived by RSM for Resistance to Wind tunnel.

Exp.	Optimal conditions				Wind Erosion	
	*D*	*C*	*B*	*A*	Experimental (%)	Predicted (%)
1	13.00	18.90	17.70	8.18	91.40	91.51
2	10.99	14.05	14.20	8.27	90.90	91.00
3	14.08	17.65	20.77	8.44	90.80	91.00

The results showed that the difference between the percentages of predicted data and calculated data to optimize mulch mixture for the values of penetrometer and wind erosion were low which shows a high accuracy of the software in this research (there was below 2% difference). The results for optimization to create a resistant layer against penetration showed that Black Strap creates a resistant compound, and as Black Strap is used less in the compound, the values for Biosolids and Chaff are increased so that it can rise soil resistance. According to the optimization results, Black Strap was the most efficient mulch mixture to increase the surface resistance. However, the results for reducing wind erosion also showed that it is required to use constant value of Chaff in the mulch because of its properties (clods, and size of particles). Also, Black Strap creates a resistant layer, and by decreasing Black Strap, Biosolids must be used more in order to increase the resistance of mulch against erosion. The optimization results in using the optimum mixture has been really significant so that the determined result could be obtained by mixing appropriate values of given materials.

## Conclusion

In this study, a central compilation scheme is developed in laboratory conditions to create organic mulch. According to the results of the analysis, the optimum conditions for the compound mulch for compression of 2 kg/cm^2^ were 11.50 g Black Strap, 16.60 g Biosolids, 18.33 g Manure, and 4.89 g Chaff. ANOVA analyzes expresses the accuracy of the statistical importance of the model in connection with the high F-value (26.241), low P-value (<0.0001), R^2^ (0.9541%), and adequate precision (21.521). Also, optimum conditions of mixed mulch for wind erosion (17 g/m^2^ h) were obtained 13.00 g Black Strap, 18.90 g Biosolids, 17.70 g Manure, and 8.18 g Chaff. The ANOVA expresses the accuracy of the statistical importance of the model in relation to the high F-value (59.069), low P-value (<0.0001), R^2^ (0.9710%), and adequate precision (30.514). Due to the fact that the optimum components presented by the software are re-checked and there is no difference between the results, simulation results can be expressed reliably. Given that these compounds are blended with water and the particles of the sand are dissolved rapidly with these compounds, a follicular state occurs on the surface of the soil and forms a suitable crust. This crust is an important factor in reducing wind erosion.

## Data Availability

The experimental data used to support the findings of this study are included in the article and readers can access it through the article content. Raw data regarding the findings and any other information can be requested from the corresponding author of the paper via e-mail.
